# SAM: A Unified Self-Adaptive Multicompartmental Spiking Neuron Model for Learning With Working Memory

**DOI:** 10.3389/fnins.2022.850945

**Published:** 2022-04-18

**Authors:** Shuangming Yang, Tian Gao, Jiang Wang, Bin Deng, Mostafa Rahimi Azghadi, Tao Lei, Bernabe Linares-Barranco

**Affiliations:** ^1^School of Electrical and Information Engineering, Tianjin University, Tianjin, China; ^2^College of Science and Engineering, James Cook University, Townsville, QLD, Australia; ^3^School of Electronic Information and Artificial Intelligence, Shaanxi University of Science and Technology, Xi’an, China; ^4^Microelectronics Institute of Seville, Seville, Spain

**Keywords:** spike-driven learning, spiking neural network (SNN), working memory, meta-learning, dendritic processing, neuromorphic computing

## Abstract

Working memory is a fundamental feature of biological brains for perception, cognition, and learning. In addition, learning with working memory, which has been show in conventional artificial intelligence systems through recurrent neural networks, is instrumental to advanced cognitive intelligence. However, it is hard to endow a simple neuron model with working memory, and to understand the biological mechanisms that have resulted in such a powerful ability at the neuronal level. This article presents a novel self-adaptive multicompartment spiking neuron model, referred to as SAM, for spike-based learning with working memory. SAM integrates four major biological principles including sparse coding, dendritic non-linearity, intrinsic self-adaptive dynamics, and spike-driven learning. We first describe SAM’s design and explore the impacts of critical parameters on its biological dynamics. We then use SAM to build spiking networks to accomplish several different tasks including supervised learning of the MNIST dataset using sequential spatiotemporal encoding, noisy spike pattern classification, sparse coding during pattern classification, spatiotemporal feature detection, meta-learning with working memory applied to a navigation task and the MNIST classification task, and working memory for spatiotemporal learning. Our experimental results highlight the energy efficiency and robustness of SAM in these wide range of challenging tasks. The effects of SAM model variations on its working memory are also explored, hoping to offer insight into the biological mechanisms underlying working memory in the brain. The SAM model is the first attempt to integrate the capabilities of spike-driven learning and working memory in a unified single neuron with multiple timescale dynamics. The competitive performance of SAM could potentially contribute to the development of efficient adaptive neuromorphic computing systems for various applications from robotics to edge computing.

## Introduction

The fast and robust working memory is a fundamental ability of the brain, which has been extensively explored by neuroscientists due to its vital roles in cognition (Nakazawa et al., 2003; Mizuseki et al., 2012; Alme et al., 2014; Taghia et al., 2018). In the field of neuroscience, research findings have revealed that working memory is the capability of maintaining and manipulating information over short time periods, which plays a vital role in accomplishing many cognitive tasks (Goldman-Rakic, 1995; Fuster, 1997). This ability of the brain to include working memory in their learning has been mimicked in artificial intelligence and deep learning, e.g., through the long short term memory (LSTM) model and its variants (Pulver and Lyu, 2017). In deep learning, one can explain the neural mechanisms and learning processes for performing complicated tasks (Vogt, 2018). However, deep neural networks are putative to be power-hungry and highly computationally intensive, which renders them unsuitable for low-power systems with online learning capability. Although efforts have been invested to solve these problems, there is still a huge gap in efficiency and cognitive abilities between the current deep learning models and their biological counterparts. As a result, it is in high demand to develop brain-inspired, energy-efficient spiking neural networks (SNNs) that may bring us closer to the capabilities of the brain in learning with working memory.

Inspired by the findings in experimental neuroscience, SNNs are presented to harness the advantages of biological neural systems (Dora et al., 2018; Liu and Yue, 2018; Yu et al., 2018, 2020; Lobo et al., 2020; Wang et al., 2021). In addition, the concept of SNNs facilitate the development of neuromorphic systems, such as Loihi (Davies et al., 2018), LaCSNN (Yang et al., 2018a), Tianjic (Pei et al., 2019), and Braindrop (Neckar et al., 2018). In order to reproduce the dynamical characteristics of biological neurons in processing spikes, various spiking neuron models such as Hodgkin-Huxley (Hodgkin and Huxley, 1952; Izhikevich, 2003), leaky integrate-and-fire (LIF) (Burkitt, 2006), and spike response model (Gerstner, 2008) are developed. These models differ greatly in incorporating the biological details of neural dynamics. Nevertheless, most SNNs use the point neuron models, which are not able to take advantage of the neuronal morphological properties, such as dendritic non-linear processing. Neuroscience research has revealed that the spiking activities of neural dendrites can change the integration of synapses (Llinás et al., 1968; Wei et al., 2001; Sjostrom et al., 2008), providing dendrites with powerful network-level computational capabilities. For example, neural dendrites play significant roles in coincidence detection and temporal sequence detection (Larkum et al., 1999; Poirazi and Papoutsi, 2020). In addition, physiological experiments have shown that dendritic signals play critical roles in brain functions, such as spatial navigation, perception processing, integration of sensory and motor input, and motor learning (Fu et al., 2012; Lavzin et al., 2012; Xu et al., 2012; Takahashi et al., 2016). Therefore, researchers have begun to focus on the application of dendritic computing in SNN modeling. Guerguiev et al. (2017) presented an SNN learning model with segregated dendrites and applied it to classification of the MNIST dataset. Urbanczik and Senn (2014) presented an SNN model based on learning with dendritic spike prediction. Their work provides a novel three-factor learning rule based on dendritic spiking activities. Many other works have also shown that dendritic processing can be fundamental to SNN modeling for efficient information coding and learning (Bar-Ilan et al., 2013; Lovett-Barron et al., 2014; Urbanczik and Senn, 2014; Schiess et al., 2016; Bono and Clopath, 2017; Guerguiev et al., 2017; Haga and Fukai, 2018). Detorakis et al. (2018) presented the neural and synaptic array transceiver (NSAT), a neuromorphic computational framework for efficient and flexible embedded learning with spiking dendrites. It can support different kinds of tasks, such as event-based deep learning, event-based contrastive divergence for supervised learning and voltage-based learning rule for sequence learning. Therefore, a new comprehensive model may include dendritic processing.

In addition, previous SNN models mostly rely on learning algorithms and ignore the intrinsic adaptability of spiking neurons. One of the most essential internal self-adaptive neuronal mechanisms is spike-frequency adaption, which plays an essential role in various types of cognitive functions, especially working memory (Fitz et al., 2020). Spike-frequency adaption, which reduces the excitability of a spiking neuron, may enhance the computational power of SNNs by endowing them with short-term memory capability. Although deep learning models with memory, such as LSTM, have shown great learning performance (Zia and Zahid, 2019), they are hand-crafted and cannot explain how biological networks in human brain can achieve very high performance on cognitively demanding tasks that require integration of neural information processing from the recent past into current processing. Due to the importance of having working memory for learning complex tasks, Bellec et al. (2018) presented the LSNN model with long short-term working memory capability for spike-based learning to learn, which uses spike-frequency adaptation for threshold adjustment along with learning process.

In this article, we present a novel biologically plausible spiking neuron model, called self-adaptive multicompartment (SAM), which integrates non-linear dynamics of spiking neurons with two important aforementioned features, i.e., neural self-adaption and dendritic non-linear processing capability. In addition, SAM includes sparse coding and spike-driven learning, which are two other important features of biologically plausible neural network models. Sparse coding enables low-power computation in the brain, and is promising to deliver the same advantage in neuromorphic systems. Spike-driven learning uses the timing and rate of spikes to govern synaptic weight changes, which is believed to be the way in which information is processed in the brain.

We apply SAM in a simple recurrent SNN architecture for a series of learning tasks, including supervised learning of the MNIST dataset using sequential spatiotemporal encoding, noisy spike pattern classification, sparse coding during pattern classification, spatiotemporal feature detection, meta-learning with working memory applied to a navigation task and the MNIST classification task, and working memory for spatiotemporal learning. The main contributions of this work are as follows:

1) A novel neuronal model, SAM, is introduced for efficient learning in SNN architectures. Due to its simplified computational integrate-and-fire form, SAM can be efficiently implemented in both software and hardware.
2) We propose a sparse, SAM-based recurrent SNN architecture, along with spike-driven learning algorithms in supervised and meta-learning frameworks.
3) The learning performance of the SAM-based SNN model is evaluated on a broad range of learning tasks, including classification of the sequential MNIST dataset, spatiotemporal spike pattern classification, spatiotemporal feature detection, meta-learning in agent navigation tasks, and meta-learning in MNIST classification.
4) We demonstrate that SAM has spatiotemporal working memory for a store and recall task, which has been shown in a few previous SNN models (Pals et al., 2020; Kim and Sejnowski, 2021).

Overall, the proposed SAM model provides a new perspective for better understanding the computational principles of learning with working memory in human brain, especially from the neuron-level point of view. Such understanding is helpful for bridging the gap between microscopic neuronal level and macroscopic network level in the field of neuroscience.

The remainder of this article is structured as follows. Section “Materials and Methods” introduces SAM, as well as the proposed SNN architecture and learning algorithm. Section “Experimental Results” presents the experimental results. And finally, the discussions and conclusions are proposed in sections “Discussion” and “Conclusion,” respectively.

## Materials and Methods

### Self-Adaptive Multicompartment Neuron Model

**FIGURE 1:**
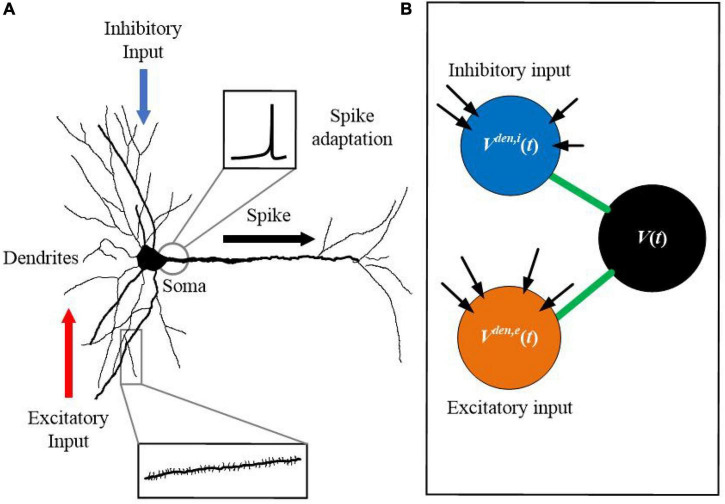
Morphological structure of a neuron with dendrites, which inspires the design of the SAM model. **(A)** Biological neuron with dendrites. **(B)** The SAM neuron model.

Previous studies have revealed that the precise timing and location of active dendrites can significantly influence neuronal functions. While dendritic excitation can drive action potential spiking, dendritic inhibition serves as an opposing force to gate excitatory activities (Grienberger et al., 2017; Muñoz et al., 2017; Poleg-Polsky et al., 2018; Ranganathan et al., 2018). Figure 1A shows the morphological structure of a biological neuron, in which dendrites deliver excitatory and inhibitory inputs from different paths, simultaneously. Figure 1B shows the proposed SAM neuron model. The soma of the neuron has the spike adaptation mechanism, which can vary the threshold according to the neuron’s firing pattern. Inspired by this morphological structure of biological neurons, we proposed the SAM neuron model. SAM has three compartments, including two dendrite and one soma compartments. It utilizes the spatial layout of different dendritic compartments to receive excitatory and inhibitory inputs. It also uses dendritic and somatic compartments to receive and send spikes, respectively. Compared with conventional point neuron models, the non-linear information processing capability of the active dendrites in SAM can enhance the learning capability of the low-resolution synaptic patterns (Cazé and Stimberg, 2020).

The membrane potentials of soma and dendrite compartments evolve using the following formulas:


(1) $$\left\{ \begin{array}{c} \tau_{v}{\dot{V}}_{j}\left(t\right)=-V_{j}\left(t\right)+R_{m}I_{j}\left(t\right)+g^{inh}\left(V_{j}^{den,i}\left(t\right)-V^{inh}\right)\\ +g^{exc}\left(V_{j}^{den,e}\left(t\right)-V^{exc}\right)\\ \tau_{v}{\dot{V}}_{j}^{den,i}\left(t\right)=-V_{j}^{den,i}\left(t\right)+R_{m}^{i}I_{j}^{i}\left(t\right)\\ \tau_{v}{\dot{V}}_{j}^{den,e}\left(t\right)=-V_{j}^{den,e}\left(t\right)+R_{m}^{e}I_{j}^{e}\left(t\right) \end{array}\right.$$

where *τ_*v*_* represents the membrane time constant and *R*_*m*_ represents the membrane resistance of soma. $R_{m}^{e}$ and $R_{m}^{i}$represent the membrane resistance of the excitatory and inhibitory dendrites, respectively. Variables *V*(*t*), *V^den,i^*(*t*), and *V^den,e^*(*t*) are the membrane potentials of soma, inhibitory dendrite and excitatory dendrite, respectively. The parameters *g^inh^* and *g^exc^* represent the synaptic conductance of inhibitory and excitatory dendrites, respectively. The parameters *V^exc^* and *V^inh^* represent the reversal membrane potential of excitatory and inhibitory dendrites, respectively. Neuron index *j* represents the *j*th neuron to be updated, and it emits a spike at time *t* when it is currently not in a refractory period.

The input current, *I*_*j*_(*t*), is defined as the weighted sum of spikes from external inputs and other neurons as:


(2) $$\left\{ \begin{array}{c} I_{j}\left(t\right)=\sum_{j=1}^{n}W_{ij}^{in}\alpha_{i}\left(t-d_{ij}^{input}\right)+\sum_{j=1}^{n}W_{ij}^{rec}\beta_{i}\left(t-d_{ij}^{rec}\right)\\ I_{j}^{i}\left(t\right)=\sum_{j=1}^{n}W_{ij}^{iin}\alpha_{i}\left(t-d_{ij}^{iinput}\right)+\sum_{j=1}^{n}W_{ij}^{irec}\beta_{i}\left(t-d_{ij}^{irec}\right)\\ I_{j}^{e}\left(t\right)=\sum_{j=1}^{n}W_{ij}^{ein}\alpha_{i}\left(t-d_{ij}^{einput}\right)+\sum_{j=1}^{n}W_{ij}^{erec}\beta_{i}\left(t-d_{ij}^{erec}\right) \end{array}\right.$$

where, $W_{ij}^{in}$, $W_{ij}^{iin}$, and $W_{ij}^{ein}$represent the synaptic weights of soma, inhibitory dendrite and excitatory dendrite, respectively. $W_{ij}^{rec}$, $W_{ij}^{erec}$, and $W_{ij}^{irec}$, on the other hand, represent the recurrent synaptic weights of soma, excitatory dendrite and inhibitory dendrite, respectively. The constants $d_{ij}^{input}$, $d_{ij}^{iinput}$, $d_{ij}^{einput}$, $d_{ij}^{rec}$, $d_{ij}^{irec}$, and $d_{ij}^{erec}$ represent the delays of input and recurrent synapses for soma, inhibitory dendrite, and excitatory dendrite. The spike trains α_*i*_(*t*) and β_*i*_(*t*) are modeled as sums of Dirac pulses, which represent the spike trains from input neurons and neurons with recurrent connections, respectively.

We discretize the SAM model with a time step Δ*t* = 1 ms. The neural dynamics in discrete time can be, therefore, formulated as:


(3) $$\left\{ \begin{array}{c} V_{j}\left(t+\Delta t\right)=\mu V_{j}\left(t\right)+\left(1-\mu \right)R_{m}I_{j}\left(t\right)+\left(1-\mu \right)V_{j}^{den,i}\left(t\right)\\ +\left(1-\mu \right)V_{j}^{den,e}\left(t\right)-\Gamma_{j}\left(t\right)z_{j}\left(t\right)\Delta t\\ V_{j}^{den,e}\left(t+\Delta t\right)=\mu V_{j}^{den,e}\left(t\right)+\left(1-\mu \right)R_{m}^{e}I_{j}^{e}\left(t\right)\\ V_{j}^{den,i}\left(t+\Delta t\right)=\mu V_{j}^{den,i}\left(t\right)+\left(1-\mu \right)R_{m}^{i}I_{j}^{i}\left(t\right) \end{array}\right.$$

where, μ = *exp*⁡(−Δ*t*/τ_*v*_). Variable *z*_*j*_(*t*) represents the spike train of neuron *j* assuming values in {0, 1/Δ*t*}. The dynamics of Γ_*j*_(*t*), representing the firing rate of neuron *j*, is changed with each spike, and is defined as:


(4) $$\Gamma_{j}\left(t\right)=\tau_{j}^{0}+\eta \cdot \tau_{j}\left(t\right)$$

where, η represents a constant that scales the deviation *τ_*j*_*(*t*) from the baseline *τ_*j*_*^0^. The variable *τ_*j*_*(*t*) can be formulated as:


(5) $$\tau_{j}\left(t+\Delta t\right)=\lambda_{j}\tau_{j}\left(t\right)+\left(1-\lambda_{j}\right)z_{j}\left(t\right)$$

where, λ_*j*_ = *exp*⁡(−Δ*t*/τ_*a*,*j*_) and *τ_*a,j*_* represents the adaptation time constant. If *V*_*j*_(*t*) > Γ_*j*_(*t*), the SAM neuron omits a spike. The parameter values of the proposed SAM model are listed in Table 1.

**TABLE 1:** Parameter settings of the SAM model.

Parameter	Value	Parameter	Value
*R*_*m*_	1 Ω	$R_{m}^{e}$,$R_{m}^{i}$	1 Ω
*τ_*v*_*	20 ms	*V^inh^*, *V^exc^*	0 mV
*d^input^*, *d^iinput^*, *d^einput^*	5 ms	*d^rec^*, *d^irec^*, *d^erec^*	5 ms
η	1.8	*τ^0^*	0.01
*τ_*a*_*	700 ms	*g*^inh^*, g*^exc^**	1 nS

### Self-Adaptive Multicompartment in Spiking Network Architecture

**FIGURE 2:**
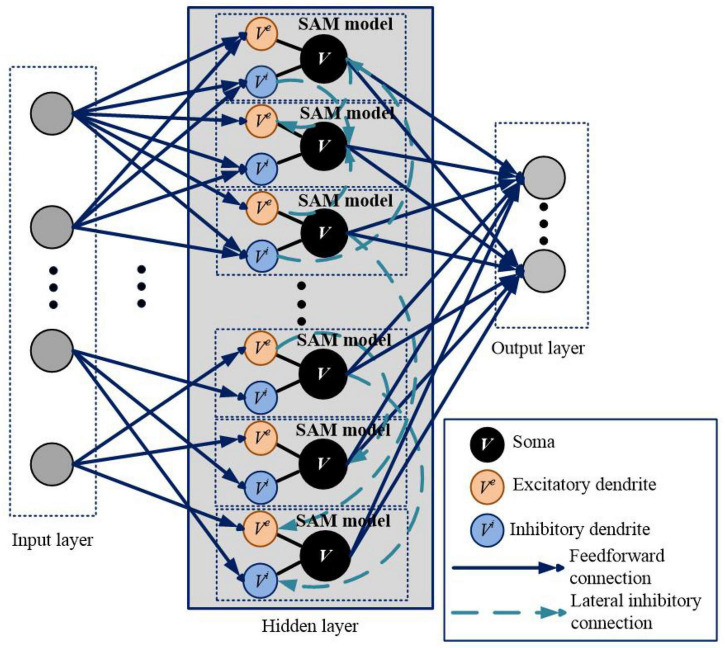
Network architecture for learning and memory based on the proposed SAM model. This architecture is comparable to a two-layer network of point neurons. In the hidden layer, dendrites and soma of different neurons are randomly connected with lateral inhibitory synapses. The gray circles in the input and output layers represent the input and output spiking neurons, respectively, which are not SAM neurons. The input and output coding styles are determined based on the tasks, which will be described in the section of experimental results.

We apply SAM in a spiking network architecture and test it in various types of learning tasks. The network architecture based on the SAM model is illustrated in Figure 2. It contains three layers, including input layer, hidden layer and output layer. The encoding schemes of the input and output layers are selected according to the task to be performed. The blue solid lines and green dotted lines represent feedforward and lateral inhibitory synaptic connections, respectively. In the hidden layer, dendrites and soma of different neurons are connected with lateral inhibitory synapses, randomly and sparsely. Dendrites of the SAM model receive the neural information from the input layer, and soma of the SAM model outputs the spikes to the output layer.

In the proposed SAM-based SNN architecture, the initial network weights are set based on a Gaussian distribution *W*_*ij*_∼$\frac{w_{0}}{\sqrt{n_{in}}}N\left(0,1\right)$, where *n*_*in*_ represents the number of spiking neurons in the considered weight matrix. *N*(0,1) represents the zero-mean unit-variance Gaussian distribution, while *w*_0_ = Δ*t*/*R*_*m*_ represents a weight-scaling factor that depends on the membrane resistance *R*_*m*_ and Δ*t*. This scaling factor is used to initialize the proposed network with a realistic firing rate needed for efficient training.

A deep rewiring algorithm is utilized since it can maintain the sign of each synapse during learning (Bellec et al., 2017). The sign is therefore inherited from the initialization of the network weights. As a result, an efficient weight initialization for the given fractions of inhibitory and excitatory neurons is required. To do so, a sign *k*_*i*_∈{−1,1} is generated randomly for neuron *i* by sampling from a Bernoulli distribution. In order to avoid exploding gradients, the weights are scaled to make the largest eigenvalue lower than 1. Thus, a large square matrix is generated and the required number of rows with uniform probabilities is selected. This dense matrix is then multiplied by a binary mask in order to produce a sparse matrix, as part of the deep rewiring algorithm, which maintains the level of sparse connectivity by dynamically disconnecting some synapses and reconnecting other synapses. In the deep rewiring algorithm used, the *L*_1_-norm regularization parameter is set to 0.01 and the temperature parameter is set to 0.

### Spike-Driven Learning of the Self-Adaptive Multicompartment-Based Spiking Neural Network Model

In conventional ANN models, the gradients of the loss function are obtained with respect to the network weights using backpropagation. Nevertheless, the backpropagation training method cannot be used directly in SNNs because of the non-differentiability of the spiking outputs. Gradients are required to be propagated either through continuous time or several time steps if time is discretized. For learning using the SAM model, a pseudo-derivative method is utilized as presented in previous studies (Courbariaux et al., 2016; Esser et al., 2016), which can be formulated as:


(6) $$\frac{dz_{j}\left(t\right)}{dv_{j}\left(t\right)}=k\max\left\{0,1-\left|v_{j}\left(t\right)\right|\right\}$$

where, *k* = 0.3 (typically less than 1) is a constant that can dampen the increase of back propagated errors through spikes by using a pseudo-derivative of amplitude to achieve stable performance. The variable *z*_*j*_(*t*) is the spike train of neuron *j* assuming values in {0, 1}. The variable *v*_*j*_(*t*) represents the normalized membrane potential, which is defined as:


(7) $$v_{j}\left(t\right)=\frac{V_{j}\left(t\right)-\Gamma_{j}\left(t\right)}{\Gamma_{j}\left(t\right)}$$

where, Γ_*j*_ represents the firing rate of neuron *j*. In order to provide the proposed network model with the self-learning capability required for reinforcement learning, the proximal policy optimization algorithm is utilized (Schulman et al., 2017). This algorithm is simple to implement and brings the self-learning capability. The clipped surrogate objective of proximal policy optimization is defined as *O^PPO^*(θ_*old*_,θ,*t*,*k*). The loss function with respect to θ is then formulated as:


(8) $$\begin{array}{c} L\left(\theta \right)=-\frac{\sum_{k<K}\sum_{t<T}O^{PPO}\left(\theta_{old},\theta ,t,k\right)}{KT}\\ +\mu_{\text{f}}\frac{1}{n}\sum_{j}{||\frac{\sum_{k,t}z_{j}\left(t,k\right)-f^{0}}{KT}||}^{2} \end{array}$$

where, *f*^0^ represents a target firing rate of 10 Hz and μ_*f*_ is a regularization hyperparameter. Variables *t*, *k*, and θ represent the simulation time step, total number of epochs, and the current policy parameter as defined in the previous research (Schulman et al., 2017). For each training iteration, *K* = 10 episodes of *T* = 2,000 time steps are generated with a fixed parameter θ*_*old*_*, which is the vector of policy parameters before the update as described in Schulman et al. (2017). In each iteration, the loss function *L*(θ) is minimized with one step of the ADAM optimizer (Kingma and Ba, 2014). This yields an instantaneous weight change of the form:


(9) $$\Delta W=\sum_{k<K}\sum_{t<T}\frac{\partial L\left(\theta \right)}{\partial W_{ij}^{x}}$$

where $W_{ij}^{x}$ represent {$W_{ij}^{in}$, $W_{ij}^{iin}$, $W_{ij}^{ein}$, $W_{ij}^{rec}$, $W_{ij}^{erec}$, $W_{ij}^{irec}$}.

We apply the reinforcement learning capability of the SAM-based SNN model in an agent navigation task, as described in previous studies (Duan et al., 2016; Wang et al., 2016). An agent is required to learn to find a target in a 2D area, and to navigate to this target from random positions in this area subsequently. This task is related to the well-known neuroscience paradigm of the Morris water maze task to study learning in the brain (Richard, 1984; Vasilaki et al., 2009). In this task, information of the current environmental state, *s*(*t*), and the reward *r*(*t*) are received by the neurons in the input layer of the SAM-based network at each time step. The environmental state *s*(*t*) is represented by the coordinate of the agent’s position. The position coordinate is encoded by input neurons according to a Gaussian population rate code. A coordinate value is assigned to each neuron in the input layer with the firing rate of *r*_*max*_ exp(−100(*ξ*_*i*_-*ξ*)^2^), where *ξ*_*i*_ and *ξ* represent the actual coordinate value and the preferred coordinate value, *r*_*max*_ is set to be 500 Hz, and the instantaneous reward *r*(*t*) is encoded by two groups of input neurons. Neurons in the first group spike synchronously when a positive reward is received, and the second group spike when the SAM model receives negative reward. The output of the SAM network is represented by five readout neurons in the output layer with the membrane potentials λ_*i*_(*t*). The action vector ζ(*t*) = [ζ_*x*_(*t*), ζ_*y*_(*t*)]*^T^*, which is used to determine the movement of an agent in navigation tasks, is calculated from a Gaussian distribution with mean μ_*x*_ = tanh[λ_1_(*t*)] and μ*_*y*_* = tanh[λ_2_(*t*)], and variance Φ_*x*_ = σ[λ_3_(*t*)] and Φ*_*y*_* = σ[λ_4_(*t*)]. The output of the last readout neuron λ_5_ is calculated to predict the value function μ*_θ_* (*t*). It predicts the expected discounted sum of the future rewards Ω(*t*) = Σ_*t*’>*t*_γ*^t^*′^–*t*^>ω(*t*’), where γ = 0.99 represents the discount factor and ω(*t*’) represents the reward at time *t*’. In addition, small Gaussian noise with mean 0 and standard deviation 0.03 is added to the SAM model at each time step. Based on our experiments, adding noise improves our model’s performance in navigation.

## Experimental Results

In this section, we first examine the dynamical properties of the proposed SAM model. We consider the dynamical activities and threshold variable of the SAM model under different levels of external currents, and investigate the impacts of critical parameters on the dynamical characteristics of SAM. Additionally, we show more simulation results, including supervised learning on sequential spatiotemporal patterns in different tasks and transfer learning with memory using SAM. Next, we examine the meta-learning capability of SAM in a reinforcement learning framework for a navigation task. Finally, we explore the high-dimensional working memory capability of SAM and the effects of critical settings on memory performance.

### Dynamical Analysis of Self-Adaptive Multicompartment Model

**FIGURE 3:**
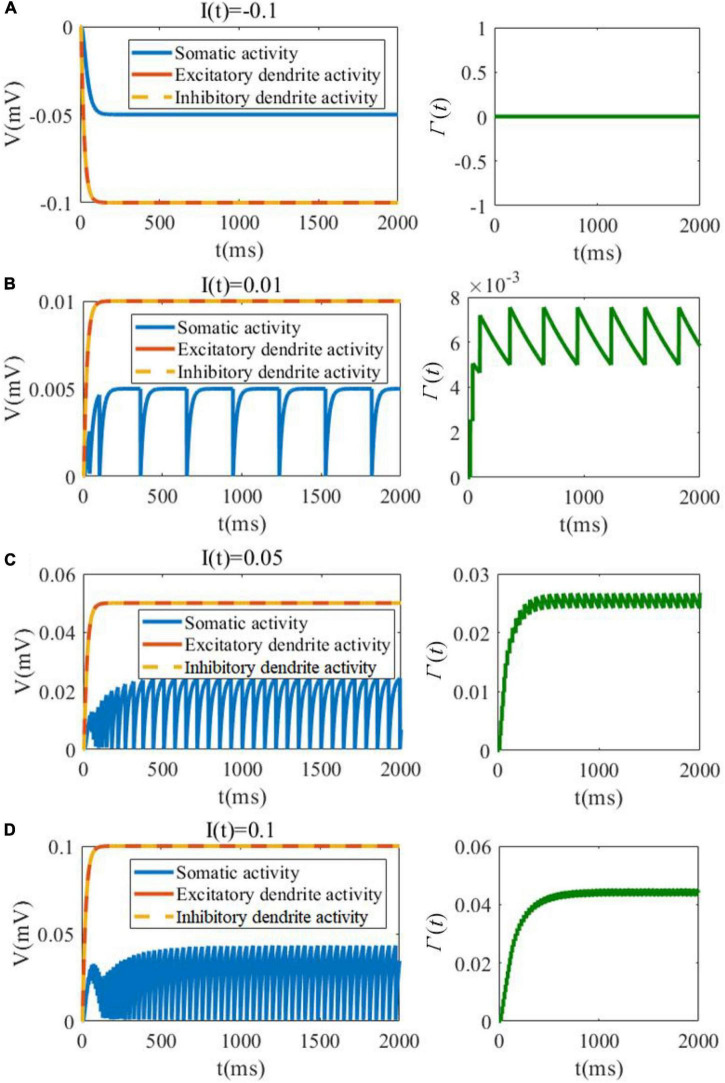
SAM membrane potential (left plots) and dynamic threshold (right plots) behavior in response to negative and low positive external currents. **(A)** *I*(*t*) = −0.1. **(B)** *I*(*t*) = 0.01. **(C)** *I*(*t*) = 0.05. **(D)** *I*(*t*) = 0.1.

In the first analytical experiment, we evaluate the evolution of membrane potentials of soma as well as excitatory/inhibitory dendrites, and investigate the variation in threshold in response to different levels of the external stimulation. As shown in Figure 3A, when the external current is negative, the somatic membrane potentials is inhibited and there is no spike and no change in the threshold variable Γ(*t*). As shown in Figures 3B–D, the dynamic changes along with the external input current increasing. It should be noted that the external current to soma only exists in the recurrent connection, and the soma cannot receive the external current from the feedforward pathway. In contrast, the dendrites can receive the feedforward external current. The detailed connection is shown in Figure 2. It is useful and meaningful for further separate the information flow between recurrent and feedforward pathways. With the increase in the input current, the amplitude and the firing rate of the neuronal soma are both increased along with Γ(*t*). The amplitude of the excitatory and inhibitory dendrites are also increased when the stimulation current is increased.

**FIGURE 4:**
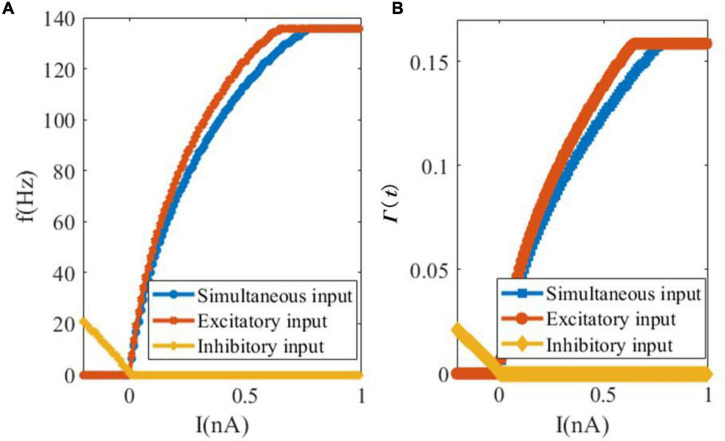
Changes of the firing rate and threshold value in response to changes in the external input currents. **(A)** Somatic firing rate. **(B)** SAM dynamic threshold Γ(*t*).

In our second analytical experiment of the dynamics of SAM, we examine the relationship between the external input current and the steady-state spiking rate or saturated threshold value, as shown in Figure 4. Figure 4A depicts the change in the firing rate of the SAM soma in response to external currents including only excitatory, only inhibitory and simultaneous excitatory and inhibitory inputs. It shows that inhibitory input depresses the firing activity of the soma, while the increase in the external excitatory current up to 0.65 nA increases the firing rate until it saturates at 135.5 Hz. The firing activity saturates at 0.77 nA when simultaneous inhibitory and excitatory currents are applied. The evolution of the dynamic threshold Γ(*t*) is also shown with respect to the change in the input currents, in Figure 4B. The saturation value of threshold Γ(*t*) equals to 0.1585 and the inflection points with excitatory and simultaneous inputs happen at 0.65 and 0.78 nA current values, respectively.

**FIGURE 5:**
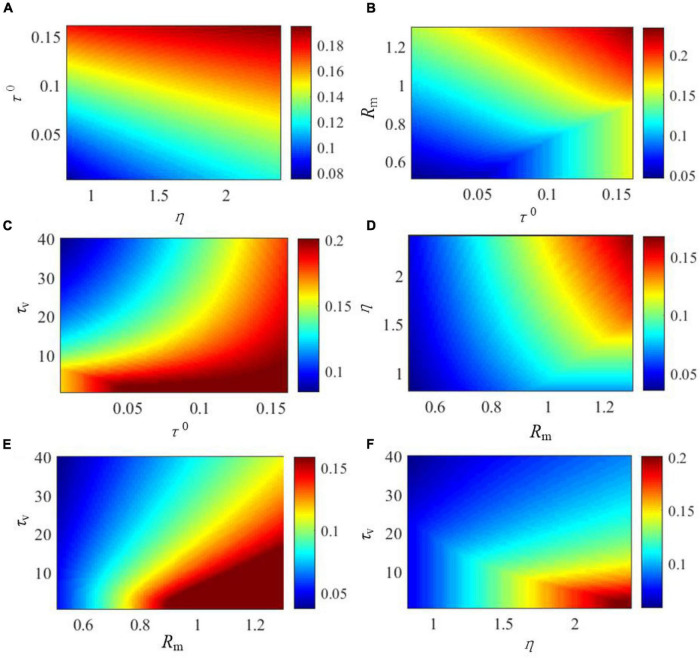
Effects of critical parameters in the SAM model on the saturated threshold value, Γ(*t*). The figures show the comprehensive effects of changes of **(A)** η and τ^0^, **(B)** *R*_*m*_ and τ^0^, **(C)** *τ_*v*_* and τ^0^, **(D)** η and *R*_*m*_, **(E)** *τ_*v*_* and *R*_*m*_, and **(F)** *τ_*v*_* and η.

**FIGURE 6:**
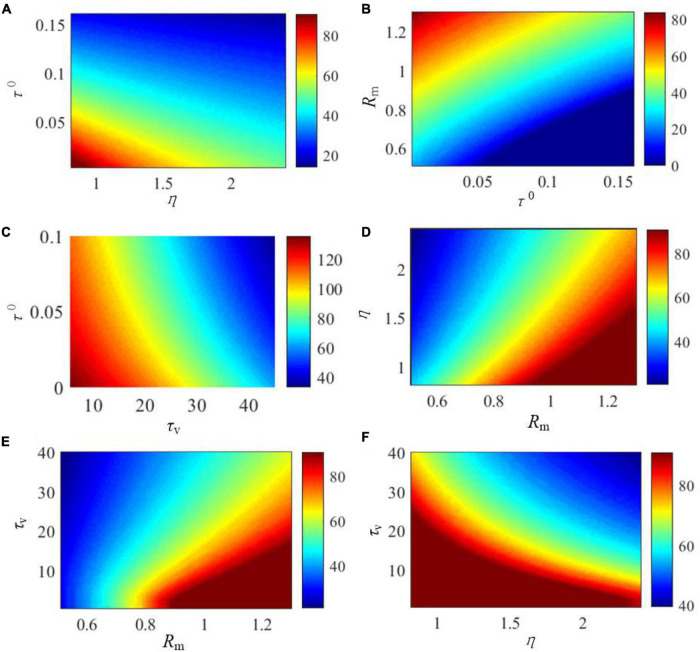
Effects of the critical parameters in the SAM model on the stable firing rate. The figures show the comprehensive effects of changes of **(A)** *R*_*m*_ and τ^0^, **(B)** *R*_*m*_ and τ^0^, **(C)** *τ_*v*_* and τ^0^, **(D)** η and *R*_*m*_, **(E)** *τ_*v*_* and *R*_*m*_, and **(F)** *τ_*v*_* and η.

The effects of the critical parameters of the SAM model on the saturated threshold value and steady-state spike frequency are further explored in Figures 5, 6. These parameters include η, τ^0^, *τ_*v*_*, and *R*_*m*_. Here, we examine SAM’s spiking behaviors by changing two of these parameters while setting the others to the standard value as listed in Table 1. Our results reveal that the increase of η, τ^0^ and *R*_*m*_ will increase the value of saturated threshold Γ(*t*) as shown in Figures 5A,B,D. Γ(*t*) reaches around 0.2 when τ^0^ = 0.15, *R_*m*_* = 1.25, or η = 2.4. When τ^0^ = 0.02, *R_*m*_* = 0.55, or η = 1, the value of Γ(*t*) will be decreased to 0.05. However, Γ(*t*) increases when *τ_*v*_* decreases as shown in Figures 5C,E,F. This means that there is a positive correlation between the saturated threshold Γ(*t*) and the critical parameters η, τ^0^, and *R*_*m*_. In addition, the relationship between the saturated threshold Γ(*t*) and *τ_*v*_* is negative. Figure 6 demonstrates the impact of the critical parameters on the steady-state spiking frequency. As shown in Figures 6A,C,F, an increase in η, τ^0^, and *τ_*v*_* decreases the spiking frequency of SAM. By contrast, the spiking frequency will increase in response to an increase in *R*_*m*_, as shown in Figures 6B,D,E, which demonstrates a positive correlation between the SAM spiking frequency and *R*_*m*_.

The above experiments on dynamical behaviors of SAM provide us with a more understanding of the mechanisms through which SAM’s intrinsic parameters control its dynamical characteristics.

### Supervised Learning of the MNIST Dataset Using Sequential Spatiotemporal Encoding

SNNs are believed to be able to produce brain-like cognitive behaviors because spikes are able to encode spatiotemporal information. By contrast, most ANN models lack timing dynamics. Here, we show how a SAM-based SNN outperforms existing ANNs on the classification of the MNIST.

In the experiments, the pixels of an image in the MNIST data are input sequentially to the SAM-based SNN in 784 time steps, because each image has 28 × 28 = 784 pixels. The input encoding method assigns a particular threshold to each input neuron that receives its corresponding pixel grayscale value. The network output is determined by the average of the output readout over 56 time steps after the digit input. The network is then trained by minimizing the cross entropy error between the label distributions and the softmax of the averaged readout.

As shown in Table 2, the mean classification accuracy of the SAM-based SNN model on MNIST outperforms the LSTM, a recurrent neural network (RNN), and a recurrent spiking neural network (RSNN) models in the previous studies (Greff et al., 2016; Huh and Sejnowski, 2017; Sherstinsky, 2020). The parameter numbers of these different models are set to keep the number of parameters in the SAM and LSTM/RNN models constant. It is worth noting that, we have not used the convolution neural network because it does not have the recurrent architecture and cannot explain and have the capability of working memory. The best performance is achieved when the SAM-based SNN model uses rewiring with a global 12% connectivity by optimizing a sparse network connectivity structure. The result suggests that only part of the parameters in the SAM-based model is finally utilized in comparison with the RNN and LSTM networks. Besides, it reveals that twice longer input duration (2 ms) has significantly lower destructive impact on the learning performance of the SAM model compared to other models. This can be attributed to the SAM model working memory capability, which can store and process more information. In addition, we conduct the ablation studies by removing the dendritic part of the model, called SAM without dendrites (SWD) model. We compare the proposed SAM model with the LSNN model presented by Bellec et al. (2018) and the SWD model. Table 2 shows that the learning accuracy of the proposed SAM model is superior to the LSNN model and the SWD model. The reasons can be summarized in two aspects. The spiking dendrites provides powerful non-linear computation capability to deal with the information flow, which is useful for the learning process. In addition, the spiking dendrites in the SAM model helps to separate the information flow in the feedfward and recurrent pathways. Therefore, this architecture solves the credit assignment problem in these two pathways during the learning process.

**TABLE 2:** Results of spatiotemporal pattern classification on sequential MNIST.

Model	Displayed time (ms)	Connectivity (%)	#Neurons	Dendrites (E/I)	Mean accuracy (%)	Maximum (%)
LSTM	1	100	128	–	79.8	98.5
LSTM	2	100	128	–	48.2	98.0
RNN	1	100	128	–	71.3	89.0
RNN	2	100	128	–	30.0	67.9
RSNN	1	12	220	–	60.9	63.3
RSNN	2	12	220	–	34.6	51.8
SWD	1	12	220	–	76.5	77.8
SWD	2	12	220	–	72.3	74.6
LSNN	1	12	220	–	94.2	94.7
LSNN	2	12	220	–	93.8	96.4
LSNN	1	100	220	–	92.0	93.3
LSNN	2	100	220	–	90.5	93.7
**SAM**	**1**	**12**	**220**	**1.0/0.6**	**95.1**	**98.4**
**SAM**	**2**	**12**	**220**	**1.0/0.6**	**94.85**	**98.4**
SAM	1	50	220	1.0/0.6	94.6	97.7
SAM	2	50	220	1.0/0.6	94.35	97.7
SAM	1	80	220	1.0/0.6	94.1	99.2
SAM	2	80	220	1.0/0.6	94.25	98.4
SAM	1	12	220	0.6/0.1	94.1	98.4
SAM	2	12	220	0.6/0.1	93.35	97.7

*The bold values means the results of this model.*

As the next step, we studied the energy efficiency of our proposed SAM-based SNN when classifying MNIST data using sequential spatiotemporal encoding. In neuromorphic hardware, only active connections in a SNN model induce a synaptic operation (SynOps). Thus, the number of SynOps for a given accuracy can be used to demonstrate the learning efficiency and potential energy consumption. It is worth noting that, one SynOps in neuromorphic systems can potentially consume significantly lower power compared to a multiply-accumulate (MAC) operation in a general purpose digital processor (Yang et al., 2021c).

**FIGURE 7:**
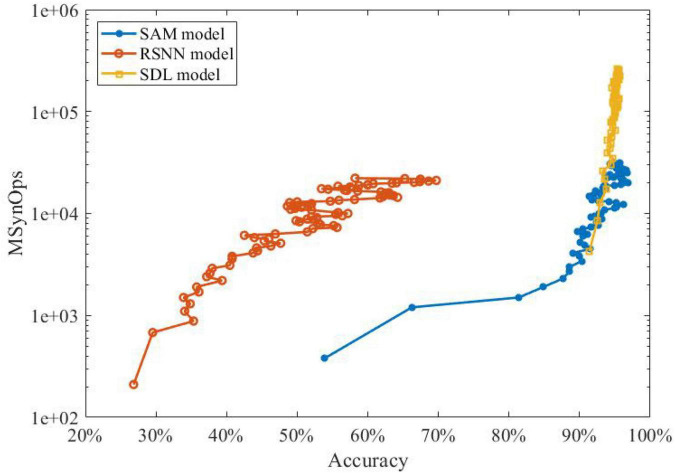
The performance investigation of learning accuracy and power efficiency of SAM compared to state-of-the-art sequential encoding methods.

Figure 7 shows that the SNN with our proposed SAM model has fewer SynOps compared to RSNN and segregated dendritic learning (SDL) model proposed by Guerguiev et al. (2017). The figure depicts that the SAM-based model can reach the same accuracy as the SDL model with almost 10M fewer SynOps. It also shows that almost 30% higher accuracy can be obtained by the SAM model in comparison with an RSNN model with the same number of SynOps. These results confirm the significant energy efficiency of our proposed SAM-based model compared to state-of-the-art models using sequential encoding for classification of the MNIST data. This is mainly because the SDL model uses a rate-based encoding strategy, while the SAM model uses a spatiotemporal approach to encoding neural information. This encoding induces fewer spikes, which helps SAM achieve a lower power consumption and better accuracy. It is worth noting that a network accuracy-based adjustment of the sensitivity in the error-coded compartment may further improve the learning accuracy of our model. In this study, however, we focused on the methodology rather than pursuing higher accuracy. Modifications and more complicated learning principles, such as adaptation of the momentum and learning rate, are left for future studies. As presented in Equations (3)–(5), the SAM model is required to compute the membrane potential and update the value, which seems to be more complicated than conventional LSTM. However, the SNN has a superior advantage, i.e., event-driven computation. It means that the spiking neurons will be triggered until the events accumulated to a certain level. The SNN model is sparsely coded, which means that the synaptic events within and communicated with the SNN model is sparse enough. In contrast, the LSTM model is frame-driven, which means the model has to update in each frame. Therefore, in previous work, Neftci et al. (2017) has presented the comparison with the number of MAC operations required for reaching a given accuracy with the number of SynOps in the spiking network for the MNIST learning task based on their dendrite-based learning algorithm for SNN with spiking dendrites. It suggests that a SynOp in dedicated hardware potentially consumes much less power than a MAC in a general purpose digital processor.

### Noisy Spike Pattern Classification

**FIGURE 8:**
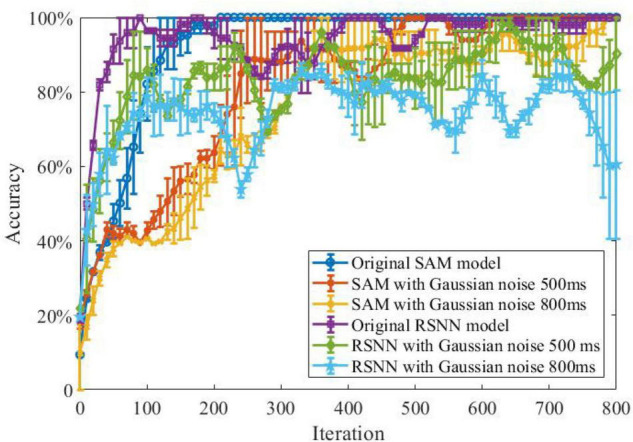
Robustness of the SAM and RSNN models on spatiotemporal spike pattern classification. We use RSNN for comparison because the proposed model is based on the RSNN architecture, with the spiking neurons replaced by the SAM neuron model. The RSNN architecture is based on reference (Pyle and Rosenbaum, 2017).

In another experiment, we study the ability of the SAM model in discriminating different spatiotemporal spike patterns. For this purpose, we designed a five-class classification task and construct one template spike pattern for each category. Each template is randomly generated and kept fixed thereafter. Each spike pattern contains neurons firing certain numbers of spikes across time. To investigate the effect of noise, spike patterns of each category are then instantiated by adding a jitter to the template pattern, which means each spike of the pattern is jittered by a random Gaussian noise with zero mean and an standard deviation of δ = 500 or 800 ms. As shown in Figure 8, SAM is much more robust than the RSNN. This is mainly because the self-adaptive mechanism of the SAM model enables more robustness during learning. In addition, the dendritic non-linearity endows the SAM neuron model with better learning performance in the presence of noise.

### Sparse Coding During Pattern Classification

**FIGURE 9:**
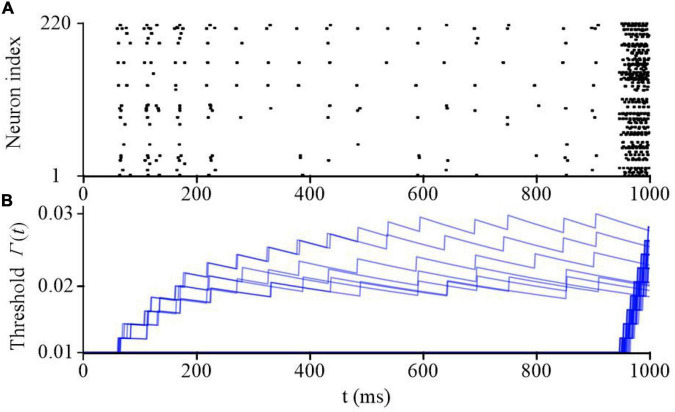
The network dynamics during sequential spatiotemporal learning task of spike patters. **(A)** The raster plot of the SAM neurons in the hidden layer. **(B)** The adaptive threshold values of the SAM model evolving with time in the hidden layer.

Sparse coding is a representative feature of SNN models. Figure 9 shows the raster plot of the proposed SAM-based SNN model in the classification task of spike patterns. These patterns are encoded by population coding through the firing probability of 80 input neurons. An additional input neuron is activated when the presentation of the spike patterns are finished to prompt an output from the SAM network. The raster plot of the somatic activities in the hidden layer, which includes 220 neurons, after training are plotted in Figure 9A when the input pattern is sequentially presented. It reveals that the SAM-based SNN model processes the neural information using very sparse neuronal spikes, which results in low power consumption on neuromorphic hardware. The dynamics of the firing thresholds of SAM neurons in the hidden layer are plotted in Figure 9B. During the learning of the sequential spike patterns, the adaptive thresholds are adjusted and stabilized at a final saturation level.

### Feature Detection Capability of Self-Adaptive Multicompartment

**FIGURE 10:**
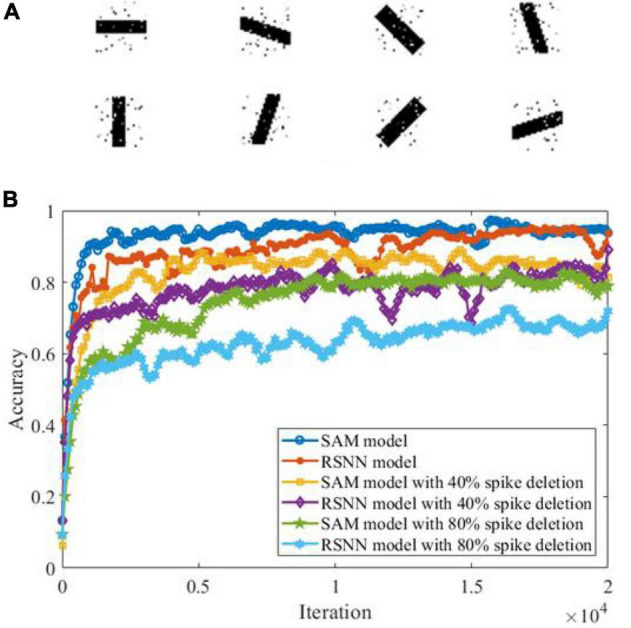
Performance analysis in a spatiotemporal feature detection task. **(A)** Images in the feature detection task. **(B)** Performance comparison between SAM and RSNN models.

Previous studies have shown that SNN models can detect features from background activities by using a spike-based processing strategy (Masquelier et al., 2008). Here, we examine SAM’s ability in a feature detection task. In this task, we created eight image patterns each representing one direction pattern, including 0°, 22.5°, 45°, 67.5°, 90°, 112.5°, 135°, and 157.5°. Each image contains 729 (27 × 27) pixels, of which 10% are randomly selected to receive Gaussian noise as shown in Figure 10A. Figure 10B shows the learning performance of the SAM and RSNN models in detecting the features in each of these patterns. The figure shows that SAM capability in spike-driven learning with memory leads to successful detection of features in different patterns. In addition, the results suggest that the SAM model is more robust in detecting patterns with features contaminated by background noise and when 40 or 80% of spikes are deleted randomly with a defined probability, to train neurons for the spike deletion noise. The performance of the proposed SAM model is significantly improved as compared to the conventional RSNN model, because the dendritic non-linear processing mechanism can better learn the information in the input spike signals. The spiking patterns are also learned and stored by the self-adaptation mechanism of the soma in the SAM model, which adapts to the spatiotemporal features with different types of noise.

### Self-Adaptive Multicompartment Model for Meta-Learning With Working Memory

In this section, we show that SAM can be used for meta-learning. We demonstrate this by applying SAM to two types of meta-learning tasks, which are meta-learning in a navigation tasks and meta-learning for MNIST classification. Both tasks require the meta-learning capability of the SNN, which means their performance depends on the guidance of the past experience.

**FIGURE 11:**
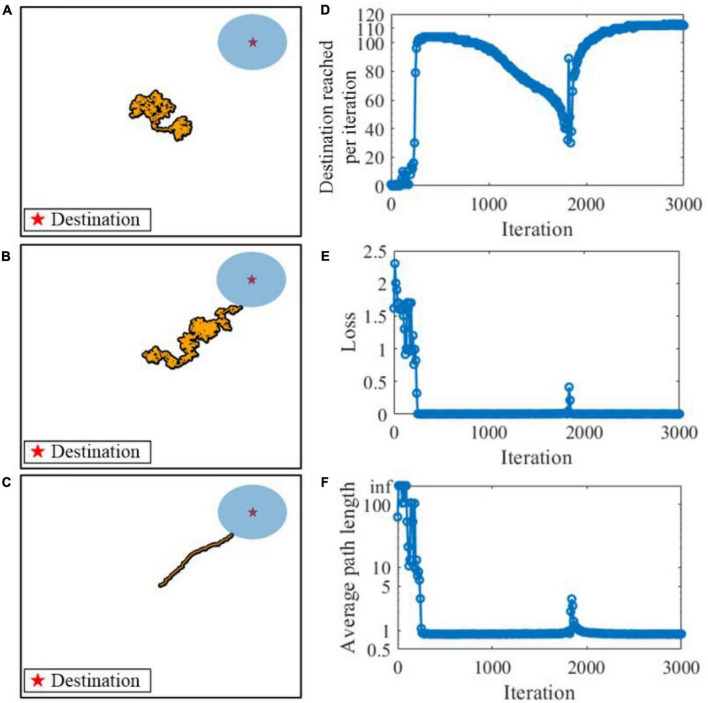
Meta-learning based on the SAM model in a navigation task. The orange points show the navigation path during the task. **(A)** Before learning. **(B)** During learning. **(C)** After learning. **(D)** Destination reached per iteration during learning. **(E)** The navigation loss function *L*(θ) during learning. **(F)** The average path length to the destination during learning.

We first consider the learning performance of the SAM model in meta-learning of a navigation task. Figure 11 shows the meta-learning capability of our proposed SAM model for flexible planning in the navigation task. A virtual agent is simulated and shown as a point, in the simulation environment of a 2D arena. The agent is controlled by the SAM-based SNN model. At the beginning of an episode and after the agent reaches a destination, the position of the agent is set randomly with a uniform probability within the search arena. At each time step, the agent selects an action by generating a small velocity vector of the Euclidean norm. After the agent reaches the destination, it receives a reward of 1. Figures 11A–C demonstrate that the SAM-based agent learns to navigate to the correct destination point after learning. Figure 11D shows the number of successful instances of the agent reaching the destination per learning iteration. Each iteration contains a batch of 10 episodes, and the weight are iteratively updated across these episodes. The figure demonstrates that the navigation performance drops between 1,000 and 2,000 iterations, and eventually reaches its max at 3,000 iterations. This trend is also shown in the navigation loss function *L*(θ), which reaches zero and remains at zero most of the time after 300 iterations, as shown in Figure 11E. Finally, the agent learns to find the shortest path to the destination after learning the navigation task, as shown in Figure 11F. A fluctuation occurs in the 1,800th iteration during training, but it quickly converges to a stable state again. This reveals that the SAM-based agent has the meta-learning capability in the navigation task because of its self-adaptive mechanism with dendritic non-linearity to process input information.

**FIGURE 12:**
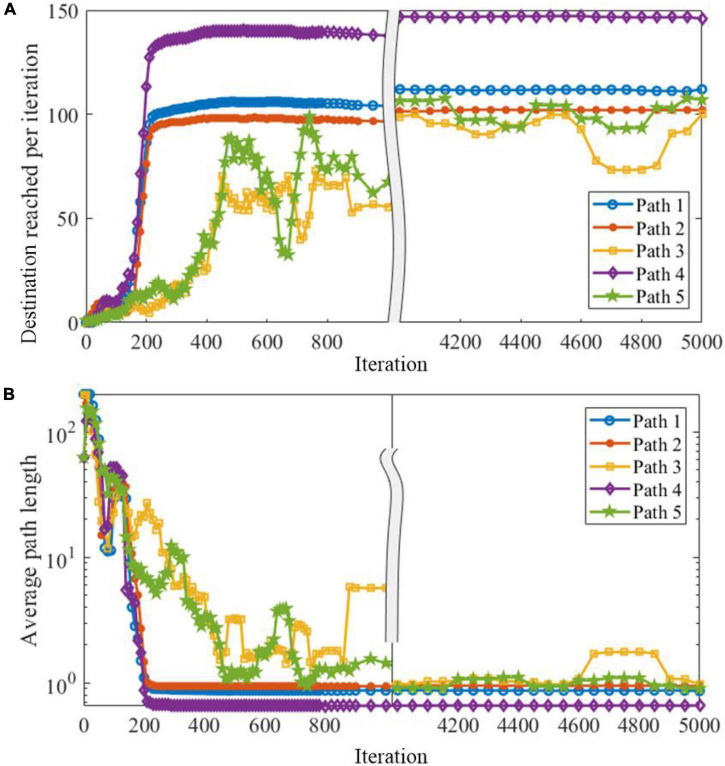
Meta-learning capability of the proposed SAM model in the test of five paths. **(A)** Destination reached per iteration in five paths. **(B)** Average path length in five paths.

Next, we investigate SAM-based network behavior before, during, and after meta-learning in another navigation task. In this task, the start and destination locations are randomly set to form five paths. The result is shown in Figure 12. Here, the objective is to maximize the number of destinations reached in each episode. For that, a family *F* of tasks is defined according to the infinite set of possible destination positions. For each episode, an optimal agent is required to explore until it reaches and memorizes the destination position, and exploits its previous knowledge to find the shortest path to the destination. The training in the first path uses back propagation through time (BPTT) (Werbos, 1990), with deep rewiring algorithm in the surrogate objective of the proximal policy optimization (PPO) algorithm. Figure 12 reveals that an agent based on the proposed SAM model is able to autonomously navigate based on its powerful meta-learning capability. Table 3 shows the comparison between SAM and LSNN model by Bellec et al. Four factors are considered to be compared, including neuron number, connectivity, goal reached number and learning convergence period. As shown in Table 3, neuron number used by SAM is lower than LSNN, and the connectivity of SAM is more sparse than LSNN. It results in the lower power consumption by SAM than LSNN. In addition, the SAM model reaches more goals than LSNN, demonstrating more powerful meta-learning capability. Convergence period of SAM is lower than LSNN, which means SAM can learn more fast in a reinforcement meta-learning framework. The reasons contain two aspects. Firstly, the non-linear computation of spiking dendrites helps to encode the precise location of the agent. Secondly, the pathway separation by the spiking dendrites of the proposed SAM model helps to deal with the credit assignment problem, therefore further improves the learning performance in the reinforcement learning framework.

**TABLE 3:** Comparison with the related SNN model.

Model	Neuron number	Connectivity	Goal reached number	Convergence period
LSNN	400	20%	25	∼300 k
SAM	220	12%	112	∼30 k

**FIGURE 13:**
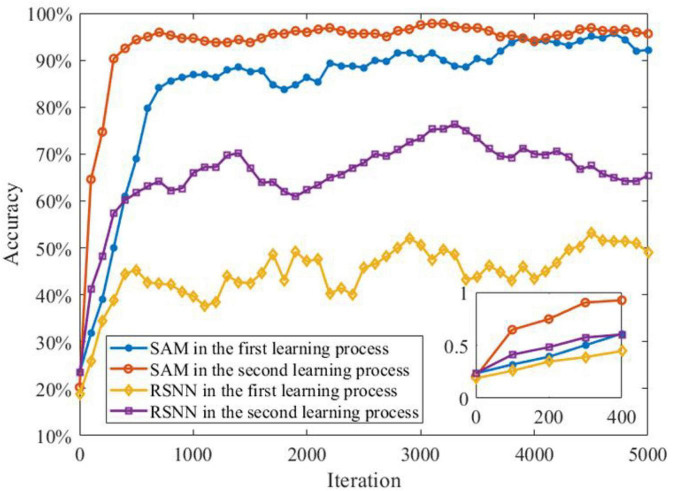
Meta-learning capability of SAM model on sequential MNIST data set.

The transfer learning capability of the SAM model is also studied for the MNIST classification. In order to demonstrate this capability, we divide the sequential MNIST training dataset into two parts for supervised learning. The first part contains 30,000 images of 0–4 digits, while the second part includes 3,000 images of 5–9 digits. In the first learning process, the first part of the MNIST data set is used to train the SAM-based SNN model. After the first learning process, the second part of the data set is used for the SAM-based SNN model training, i.e., the second learning process. Figure 13 shows that the learning performance of the second learning process is better than the first one. This can be attributed to the transfer learning capability of the SAM model. In addition, the figure shows that the SAM-based SNN model in the first and second learning processes outperforms the conventional RSNN model. The RSNN model has poor learning performance in the first learning process. In addition, higher learning speed, and convergence rate is shown in the second learning process of the SAM-based SNN model. This result suggests that the SAM-based SNN model has powerful transfer learning capability in the learning of spatiotemporal patterns.

### Working Memory for Spatiotemporal Learning

**FIGURE 14:**
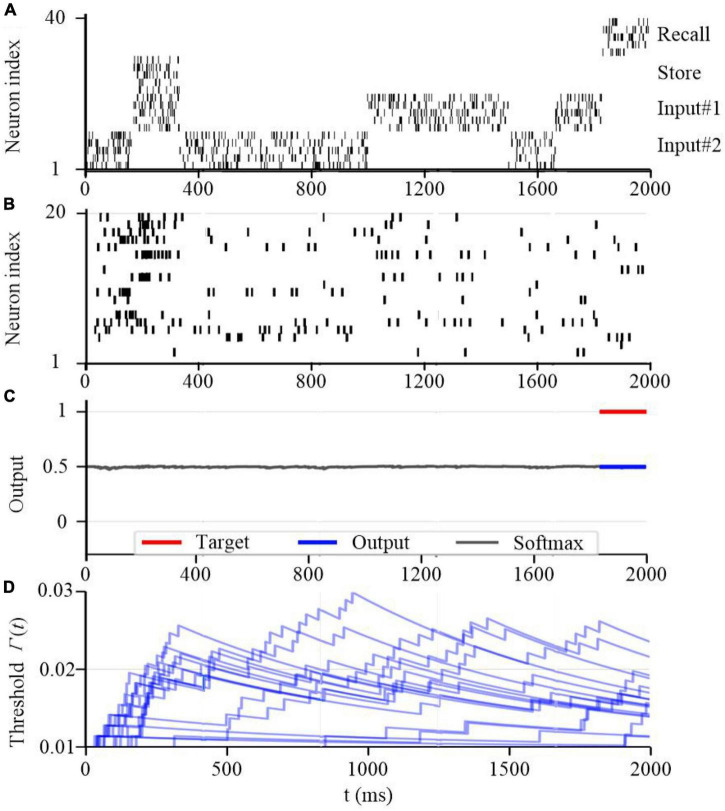
Working memory capability of the proposed SAM model at the beginning of the training process. **(A)** Spiking activities of the input neurons for the store and recall commands as well as the feature vectors #1 and #2. **(B)** Spiking activities of the SAM model in the proposed SNN architecture. **(C)** Traces of the activation of the sigmoidal readout neurons. **(D)** The temporal evolution of the firing threshold Γ_*j*_(*t*) of SAM model.

**FIGURE 15:**
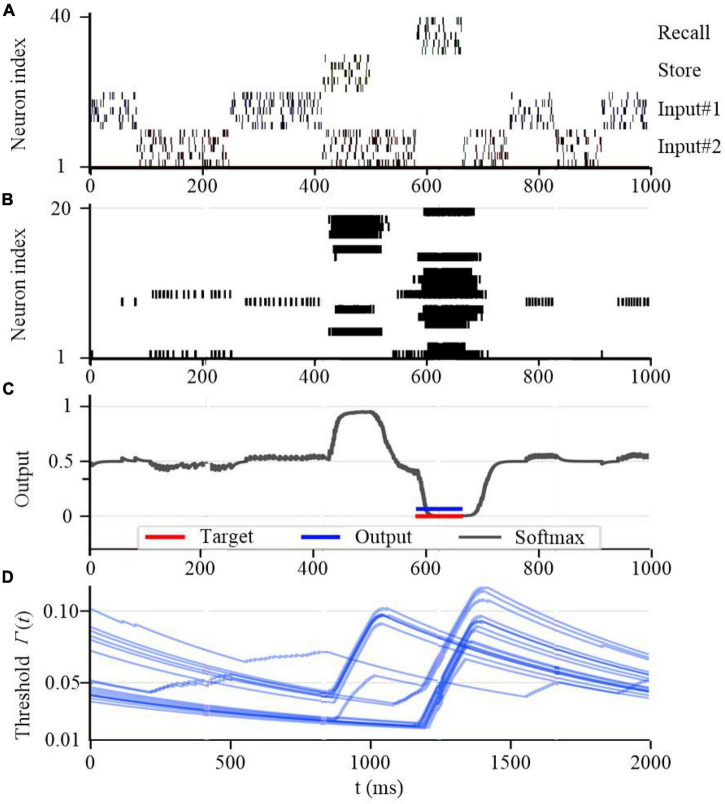
Working memory capability of the proposed SAM model after training. **(A)** Spiking activities of the input neurons for the store and recall commands as well as the feature vectors #0 and #1. **(B)** Spiking activities of the SAM model in the proposed SNN architecture. **(C)** Traces of the activation of the sigmoidal readout neurons. **(D)** The temporal evolution of the firing threshold Γ_*j*_(*t*) of SAM model.

To further test the working memory capability of the SAM model, we developed a store & recall task with working memory similar to Wolff et al. (2017). In this task, the SAM-based SNN model receives a sequence of frames, each represented by 10 train of spikes in a specific period of time, to represent input #1 and input #2 using input neurons 1–10 and 11–20 spiking activities, respectively. For instance, the first frame in input #1 consists of 10 spike trains between 200 and 350 seconds as shown in Figure 14A. Besides, the figure shows that the SAM-based SNN input neurons receive random store and recall commands using neurons 21–30 and 31–40, respectively. The store command means direct attention to a particular frame of the input stream. The goal of this task is to reproduce this frame when a recall command is received. To perform the store and recall operation, a SNN with 20 SAM neurons in its hidden layer and 20 output sigmoid neurons is trained. This is similar to previous research such as Roy et al. (2019). Figure 14 shows a sample segment of a test with the spiking activities of the SAM neurons at the beginning of the training, which reveals that the SAM-based SNN cannot accomplish the store and recall function without training. Here, Figure 14B shows random spiking activities in the 20 SAM neurons before any learning, while the activity of the sigmoidal output neurons (Figure 14C) and the threshold of the 20 SAM neurons show no specific behavior before learning starts. However, after the training process is completed and as shown in Figure 15, the SAM-based SNN can successfully realize the working memory function. In addition, it shows that the dynamic thresholds change more consistently after training in the store & recall task. This kind of working memory exhibited by the SAM model refers to the activity-silent form of working memory in human brain, which has been explored in the neuroscience study by Wolff et al. (2017).

### Critical Parameters of Self-Adaptive Multicompartment in Working Memory

**FIGURE 16:**
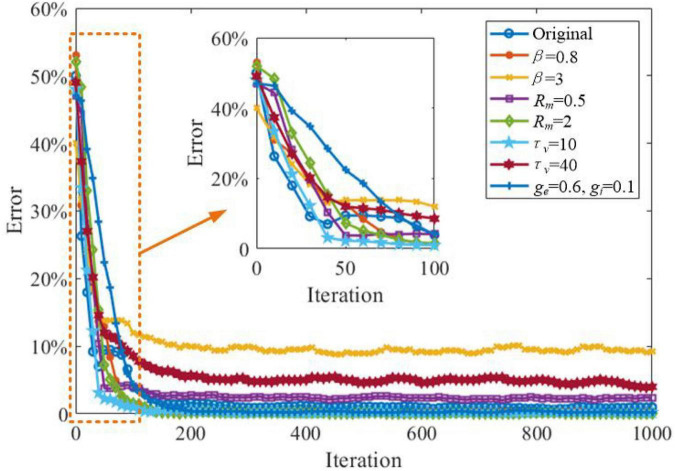
Investigation of the impacts of the critical parameters on the working memory capability of the SAM model applied to the store-recall task.

In this section, we explore the effects of critical parameters on the working memory performance of the SAM model, when applied to the store & recall task mentioned in the previous section. As shown in Figure 16, two values for β, the sum of Dirac pulses, which represent the spike trains from neurons with recurrent connections are considered as β = 0.8 and β = 3. This shows that a high value of β = 3 will decrease the working memory capability of the SAM model. In addition, high values of *τ_*v*_*, the SAM membrane constant, will also reduce the working memory performance, significantly. When the connectivity conductance of the dendrites are decreased to *g_*e*_* = 0.6 and *g_*i*_* = 0.1, the learning convergence speed is decreased compared to the original parameter settings of *g_*e*_* = 1.0 and *g_*i*_* = 0.6. The SAM model with lower values of β and higher level of the membrane resistance of soma, *R*_*m*_, will maintain the memory performance of the original settings. Finally, Figure 16 also shows the SAM model with *τ_*v*_* = 10 enhances the learning convergence speed. Since the membrane constant *τ_*v*_* has a negative correlation with the saturated threshold value and the stable spiking frequency, it reveals that a higher stable spiking frequency and a saturated threshold value of the SAM model will further improve the learning convergence speed in working memory tasks. In contrast, the lower coupling strength of the dendrites will decrease the learning convergence speed.

## Discussion

A critical open problem in computational neuroscience is to understand how brains process and learn the neural information based on not only the current situations, but also the recent past experience (Lansdell and Kording, 2019). This mechanism is related to working memory in the field of neuroscience, which has been revealed to play vital roles in perception, cognition and learning (Reinhart and Nguyen, 2019). Learning with working memory has been implemented by LSTM models in the field of artificial intelligence, but the neuron-level mechanisms underlying this formulation have not been fully explained by neuroscience because it is difficult to separate mechanisms of working memory from the computational principles of the SNN models. In this article, a novel unified spiking neuron model, named SAM, for integrating learning with memory is presented.

The proposed SAM model is motivated by two critical mechanisms in addition to the basic integrate-and-fire neural principle. These mechanisms include dendritic non-linear processing, and the self-adaptation properties of spiking neurons. For the first time, this study considers both the excitatory and inhibitory dendrites in a single neuron model, which demonstrates high performance in information processing and learning in an SNN framework. The critical self-adaptation mechanism, spike-frequency adaptation, is introduced in the soma of the SAM model. This adaptation mechanism provides the capability of long short-term memory for the proposed model.

**FIGURE 17:**
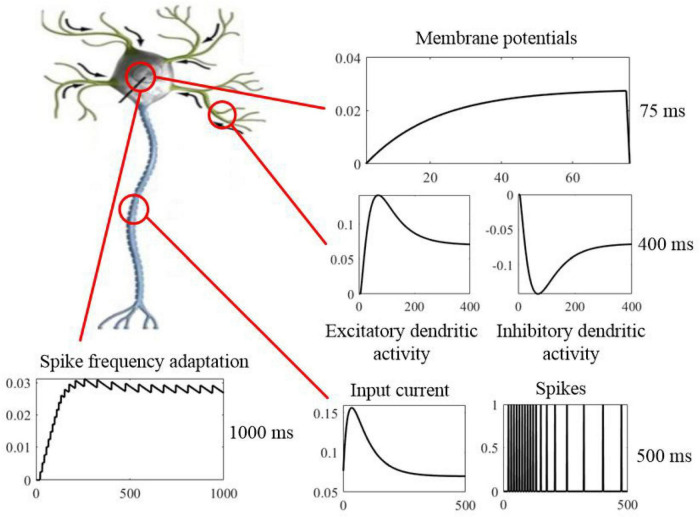
Neurobiological computation of the SAM model with working memory on multiple timescales, including short timescale of membrane potentials (top panel), medium timescale of dendritic activities and input spikes (right middle and bottom panels), and long timescale of spike frequency adaptation (left bottom panel). Sustained neural spiking activity can be considered as a computation form on short timescales. However, physiological processes of the neuronal dendrites other than the evolving somatic membrane potential provide the mechanism for information on successively longer timescales. The spike-frequency adaptation mechanism of the soma in the SAM model, has the longest timescales with slower dynamic variables. The functional distinction between memory and computation is dependent on the multiple timescales of dynamic variables.

SAM presents a cellular mechanism for working memory on short timescales, where information is stored and maintained in physiological processes that govern neural excitability as a function of experience. SAM’s membrane potential dynamics with neural adaptation mechanism have longer time constants, as shown in Figure 17. Information coded into spiking activity is encoded into slow dynamics of membrane potential, which means writing into memory. Therefore, dynamic threshold of the somatic membrane act as memory registers that store real information, and they are the physical address of the memorandum. Memory traces can persist in the absence of sustained spiking, excitatory feedback, or synaptic plasticity and are not influenced by membrane reset or the integration of new information into the membrane state. Because adaptation dynamics are coupled to the membrane potential of SAM, memory traces continuously exert an impact on the active membrane to read from memory. This kind of cycle with encoding and retrieval between coupled dynamic variables with different timescales can result in the basis of a neurobiological read-write memory (Fitz et al., 2020). That is to say, the rapidly changing membrane dynamics transforms an analog input to a binary output, and slower neural dynamics with adaptive threshold provides information storage in the working memory. Therefore, memory, computation, and learning can be integrated and implemented within a single neuron by the proposed SAM model, and the functional distinction is dependent on the multiple timescales accordingly.

Neuroscience studies have argued that the brain learns the spiking patterns in a sequential spatiotemporal form, other than the static formation of the processing strategies by conventional ANNs (Rolston et al., 2007). To examine this, we investigated the spatiotemporal learning capability of our SAM model. As shown in Table 2, SAM has superior learning accuracy on the sequential MNIST dataset with both 1 ms and 2 ms time delays. In addition, Figure 7 shows that a lower SynOps number is required by SAM, when compared to the RSNN model and the SDL model by Guerguiev et al. (2017). In fact, the SDL model is a representative work to utilize the dendritic processing in the efficient learning of SNN models. Our study demonstrates that the self-adaptation mechanism used in the soma of the SAM model, in addition to the dendritic processing that is similar to Guerguiev et al. (2017), can further cut down the number of SynOps produced by the SNN models, which could be a vital potential mechanism for the low power consumption of biological brains. Considering the significantly lower SynOps, SAM can result in lower power consumption in neuromorphic hardware.

Classification is a common task to examine the learning capability of an intelligent system. Two categories of classification tasks were used in this study, including spike pattern classification and feature detection. Besides, a robustness test was conducted in these two tasks by adding two types of noise, namely Gaussian jitter noise and spike deletion noise. As shown in Figures 8, 10, the proposed SAM model is robust to different noise types. Since one SAM neuron can be designed to produce different output spike numbers for different categories of input spike patterns, it can therefore enable a single-neuron multi-class classifier. In addition, SAM’s efficiency and robustness makes it superior to previous model as a spike-based classifier for real-world classification tasks when proper encoding strategies are used.

Recent neuroscience studies have revealed that dendritic processing is critical for spontaneous neuronal sequences for single-trial learning, pathway-specific gating, shaping neural plasticity, and fear learning (Bar-Ilan et al., 2013; Lovett-Barron et al., 2014). Bono and Clopath (2017) investigated how dendrites enable multiple synaptic plasticity mechanisms to coexist in a single neuron by implementing biologically plausible neuron models with dendritic compartments. Their findings reveal that memory retention during associative learning can be prolonged in SNNs by containing dendrites. Haga and Fukai (2018) explored the robust single-trial learning with plasticity of dendritic-targeted inhibition, which demonstrates that dendritic computation enables somatic spontaneous firing sequences for rapid and robust memory formation. Schiess et al. (2016) demonstrated that the dendrites of cortical neurons can non-linearly combine synaptic inputs by evoking local dendritic spikes, which show that non-linearity can enhance the computational power of a single neuron. Inspired by these studies, as already explained, we designed SAM to contain two dendritic compartments for excitatory and inhibitory synaptic inputs. In addition, the neuronal self-adaptation mechanism was also considered in the proposed SAM model. Previous studies have revealed that the self-adaptive threshold mechanism could contribute to the working memory of SNN models (Fitz et al., 2020). Information is encoded in spike trains and maintained within memory through sustained spiking activities that are supported by properly tuned synaptic feedback or neuronal multistability (Wang, 2001; Zylberberg and Strowbridge, 2017). By integrating both dendritic dynamics and the self-adaptation mechanism for working memory into SAM, it performed well in meta-learning in two complex tasks of agent navigation and MNIST meta-learning. As shown in Figures 11, 12, the proposed SAM model shows good performance in autonomous navigation of intelligent agents. Figure 13 further shows the meta-learning capability of SAM in MNIST classification, where the past experience of learning of the first half of the training set results in faster learning of the second half of the training set. Working memory for spatiotemporal learning is a vital mechanisms in human brain for realizing high-level cognitive functions. The spatiotemporal working memory capability of SAM model is shown in Figures 14, 15. It shows that the store-recall task can be completed by the dynamically changing membrane threshold along with BPTT learning rule.

Neuromorphic engineering is a promising approach toward artificial general intelligence. Previous studies have presented various types of neuromorphic systems (Azghadi et al., 2014, 2020), aiming at both engineering and neuroscience applications (Hodgkin and Huxley, 1952; Izhikevich, 2003; Azghadi et al., 2017; Lammie et al., 2018; Neckar et al., 2018; Yang et al., 2018a,b,c, 2019, 2021a,b; Frenkel et al., 2019; Pei et al., 2019). Due to the simple integrate-and-fire formation, the proposed SAM model can be easily implemented on neuromorphic hardware, which has the advantage of low power consumption. For instance, current implementations of neuromorphic hardware use only a few *nJ* or even *pJ* for routing a single spike. In our future research we aim to implement the SAM model on our LaCSNN neuromorphic system to apply it to various categories of applications, including embedded intelligent robots, internet of things, unmanned aerial vehicles, and edge computing.

## Conclusion

In this article, we proposed a novel neuron model, SAM, with learning and working memory capabilities. SAM implements four essential brain-inspired mechanisms, including sparse coding, dendritic non-linearity, intrinsic self-adaptive dynamics, and spike-driven learning. Experimental results showed SAM’s higher learning accuracy compared to the state-of-the-art models in supervised learning of the MNIST dataset using sequential spatiotemporal encoding, noisy spike pattern classification, sparse coding during pattern classification, and spatiotemporal feature detection. Furthermore, desired properties such as robustness, power efficiency, and meta-learning capability of SAM were demonstrated in two complex task of agent navigation, and meta-learning of MNIST classification. Working memory capability of the SAM model was also explored for spatiotemporal learning, where SAM showed great performance. In addition, we thoroughly investigated the effects of SAM’s critical parameters on its working memory performance. Due to its integrate-and-fire and spike-driven neural architecture, SAM can be conveniently implemented in neuromorphic hardware and bring high-performance learning with working memory while consuming low-power to various applications from intelligent robots, internet of things, and edge computing, to neuroscience studies investigating meta-learning and working memory.

## Data Availability Statement

The original contributions presented in the study are included in the article/supplementary material, further inquiries can be directed to the corresponding author/s.

## Author Contributions

SY developed, tested the algorithms, and wrote the manuscript from TG, JW, and BD. MA, TL, and BL-B conceptualized the problem and the technical framework. All authors contributed to the article and approved the submitted version.

## Conflict of Interest

The authors declare that the research was conducted in the absence of any commercial or financial relationships that could be construed as a potential conflict of interest.

## Publisher’s Note

All claims expressed in this article are solely those of the authors and do not necessarily represent those of their affiliated organizations, or those of the publisher, the editors and the reviewers. Any product that may be evaluated in this article, or claim that may be made by its manufacturer, is not guaranteed or endorsed by the publisher.
